# Physics-aware training for the physical machine learning model building

**DOI:** 10.1016/j.xinn.2022.100287

**Published:** 2022-07-08

**Authors:** Xuecong Sun, Yuzhen Yang, Han Jia, Jun Yang

**Affiliations:** 1 Key Laboratory of Noise and Vibration Research, Institute of Acoustics, Chinese Academy of Sciences, Beijing 100190, China; 2 University of Chinese Academy of Sciences, Beijing 100049, China; 3 State Key Laboratory of Acoustics, Institute of Acoustics, Chinese Academy of Sciences, Beijing 100190, China

**Figure 1:**
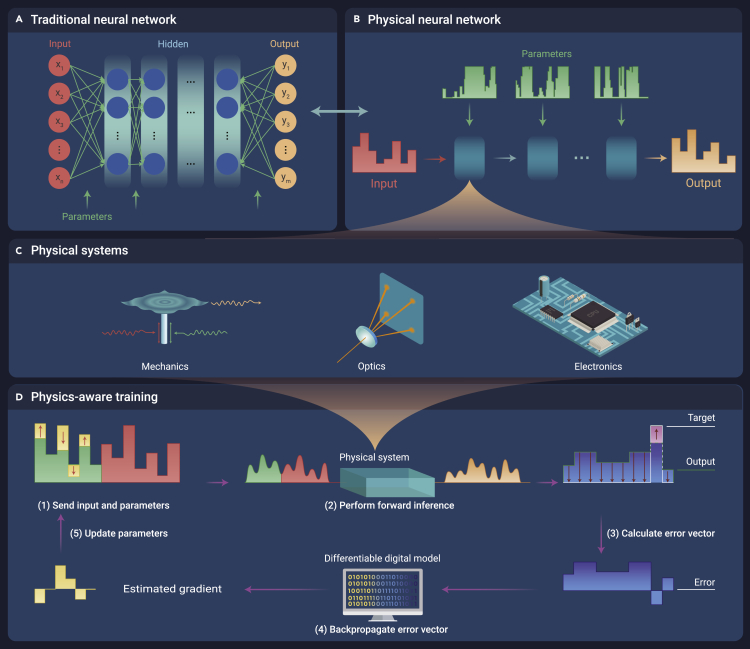
Schematics of conventional machine learning models and PNN (A and B) Diagrams of (A) a traditional neural network and (B) a PNN. (C) Three examples of controllable physical systems: mechanical, optical, and electronic. (D) Diagram of a PAT model that can be used to train virtually any controllable physical system.

In recent decades, machine learning has emerged as a very powerful computational method. Because of its exceptional successes in computer science and engineering, machine learning has ignited research interest in other disciplines, including biology, chemistry, physics, and finance. Machine learning models, which are usually regarded as mathematical models, have traditionally been implemented on the basis of digital computing platform (Figure 1A). The increasing prevalence of machine learning has been accompanied by a rapid increase of computing requirements, outpacing Moore’s law. Therefore, researchers have been committed to the development of analog computing hardware platforms to overcome the inherent limitations of computing resources. Considering that wave physics is an attractive candidate to build analog processor,^1^ wave-based analog computing platforms are emerging as an important direction to implement machine learning. Most wave-based analog processors are designed on the basis of the mathematical isomorphism between physical systems and conventional machine learning models, such as deep neural networks (DNNs),^2^^,^^3^ implying that analog processors can be trained using standard training techniques for neural networks.

However, it remains a huge challenge to design a physical system with strict operation-by-operation mathematical isomorphism, which requires a prohibitive amount of time. In fact, strict mathematical isomorphism is unnecessary to build analog computing platforms. Recently, scientists from Cornell University proposed a hybrid *in situ*/*in silico* algorithm, called physics-aware training (PAT), to train physical neural networks (PNNs) with back-propagation.^4^ PNNs are composed of layers of controllable physical systems, which lack mathematical isomorphism compared with conventional artificial neural networks. And PAT computes the forward pass on the basis of physical systems instead of training only through numerical simulations. In this way, the impact of the simulation-reality gap on model performance can be significantly reduced, and the performance penalties associated with parameter transformation from numerical simulations to real physical devices can also be avoided. Therefore, PAT allows researchers to construct PNNs from virtually any controllable physical systems and train hardware to perform desired computations. The insights gained from this study will be of great assistance to overcome the physical limitations of computing resources and render machine learning faster, more scalable, and energy efficient.

A universal framework of PNNs is shown in Figure 1B, in which the dark cyan boxes represent controllable physical systems. The input data of PNNs are usually a wave-based signal. And the parameters of PNNs correspond to some adjustable properties of the physical system, which can be trained as the weights of conventional artificial neural networks. Figure 1C shows three examples of controllable physical systems. In the audio-frequency mechanical system, input data and parameters are encoded into time-dependent forces, which can drive the voice coil of a speaker, and that in turn drives the oscillating titanium plate. In the nonlinear optical system, input data and parameters are encoded into the pulses’ spectra, which are transformed and mixed nonlinearly by passing through a crystal. In the electronic system, input data are voltage time series, and parameters are trainable scale factors of the voltage time series. The re-scaled voltage time series is then sent to the analog circuit. These systems can perform both linear and nonlinear operations, which are equivalent to common operations in conventional artificial neural networks, such as convolutions and matrix-vector multiplications. Therefore, DNN-like physical computations can be composed of various physical systems with different parameters.

Back-propagation algorithms are regarded as a key point for efficient training and good generalization of conventional artificial neural networks. The gradients of transformation in physical systems are required to apply back-propagation algorithms to PNN training. However, these gradients can only be approximated using a finite-difference approach, which makes the training slow for PNNs with a large number of parameters. To overcome the above constraints, *in silico* training that performs training within numerical simulations is adopted, whereby a differentiable digital model, $f_{model}$, is established to approximate the physical systems. Thus, both forward calculation and back-propagation can be computed quickly in simulations. In this way, the training process will be carried out solely on the computer. And then the trained parameters will be loaded into the physical systems for evaluation.

Because of errors between $f_{model}$ and the real physical system, it is difficult to directly transfer the trained parameters to real devices for expected performance. To solve this problem, a hybrid algorithm PAT is proposed, which involves computations in both physical and digital domains (Figure 1D). Specifically, the physical system is used to perform the forward pass, which can produce more accurate output than $f_{model}$ *in silico* training. And the differentiable digital model $f_{model}$ is used only in the backward pass to calculate the gradients of transformation in the physical system. The universality of PAT algorithms is demonstrated by the successful training of three PNNs composed of different physical systems (see Figure 1C). And the effectiveness of the PNNs is verified through the implementation of image and vowel classification. The experimental results show that the PNN is not only an accurate hierarchical classifier that uses each system’s unique physical transformations but also performs machine learning faster and more energy-efficiently compared with conventional electronic processors.

It should be noted that the proposed PAT can only be used to train PNNs composed of adjustable nonlinear physical systems. And it is difficult to integrate such physical systems in a small size. Thus, the stabilities and integrations of adjustable nonlinear physical structures remain critical challenges. In model training, data collection needs to be performed on the basis of the physical systems, which presents obstacles to apply parallel computing to the model training. Consequently, it will take a prohibitive amount of time to train PNNs for complex tasks using the proposed PAT. Notwithstanding these limitations, this work is of assistance to the application of DNN-based analog computing hardware platforms, particularly those in which physical data, rather than digital data, are processed or produced. PNNs perform partial computations on data within their physical domain, so smart sensors can pre-process physical information before conversion to the electronic domain (such as wave imaging and object recognizing through a multiple-scattering environment). PNNs can be further incorporated into hybrid sensing systems composed of a trainable physical front end and an all-digital machine learning-based back end.^5^ The spatial and temporal information carried by the wave fields is first encoded by the physical mechanism during the measurement process. Then the collected data can be decoded using machine learning to extract the desired information. The hybrid system can be regarded as a collaboration framework of analog computing and digital computing, which can be jointly trained through the error back-propagation between the physical front end and the digital DNN-based back end. Therefore, the physical front-end can be interpreted as a trainable layer of the machine learning model. And the jointly learned measurement and processing settings can yield considerably higher speed of operation and processing efficiency and lower power consumption, particularly when practical data originate from measurement operations with a large number of analog sensors. The hybrid sensing system will break down more barriers in conventional sensing to provide more useful information that could not be captured before.

Although this work focuses on a classification model, the proposed method can also be extended to a regression model, even to a deep reinforcement learning model. Even though the physical realizations of PNNs remain a limitation, this work opens a new approach to analog computing with a wide range of potential applications.
